# Inhibition of Microtubule Affinity Regulating Kinase 4 by Metformin: Exploring the Neuroprotective Potential of Antidiabetic Drug through Spectroscopic and Computational Approaches

**DOI:** 10.3390/molecules27144652

**Published:** 2022-07-21

**Authors:** Ghulam Md. Ashraf, Debarati DasGupta, Mohammad Zubair Alam, Saleh S. Baeesa, Badrah S. Alghamdi, Firoz Anwar, Thamer M. A. Alqurashi, Sharaf E. Sharaf, Waleed Al Abdulmonem, Mohammed A. Alyousef, Fahad A. Alhumaydhi, Anas Shamsi

**Affiliations:** 1 Department of Medical Laboratory Sciences, College of Health Sciences, and Sharjah Institute for Medical Research, University of Sharjah, Sharjah 27272, United Arab Emirates; 2 College of Pharmacy, University of Michigan, 428 Church Street, Ann Arbor, MI 48109, USA; debarati@med.umich.edu; 3 Pre-Clinical Research Unit, King Fahd Medical Research Center, King Abdulaziz University, Jeddah 21589, Saudi Arabia; mzalam@kau.edu.sa (M.Z.A.); basalghamdi@kau.edu.sa (B.S.A.); 4 Department of Medical Laboratory Sciences, Faculty of Applied Medical Sciences, King Abdulaziz University, Jeddah 21589, Saudi Arabia; 5 Division of Neurosurgery, College of Medicine, King Abdulaziz University, Jeddah 21589, Saudi Arabia; sbaeesa@kau.edu.sa; 6 Department of Physiology, The Neuroscience Research Unit, Faculty of Medicine, King Abdulaziz University, Jeddah 21589, Saudi Arabia; 7 Department of Biochemistry, Faculty of Science, King Abdulaziz University, Jeddah 21589, Saudi Arabia; firoz_anwar2000@yahoo.com or remodelinagliptingfanwar1@kau.edu.sa; 8 Department of Pharmacology, Faculty of Medicine, King Abdul-Aziz University, Rabigh 21589, Saudi Arabia; thamer@kau.edu.sa; 9 Pharmaceutical Chemistry Department, College of Pharmacy, Umm Al-Qura University, Makkah 21955, Saudi Arabia; sesharaf@uqu.edu.sa; 10 Clinical Research Administration, Executive Administration of Research and Innovation, King Abdullah Medical City in Holy Capital, Makkah 24246, Saudi Arabia; 11 Department of Pathology, College of Medicine, Qassim University, P.O. Box 6655, Buraydah 51452, Saudi Arabia; dr.waleedmonem@qu.edu.sa; 12 Division of Neurosurgery, College of Medicine, King Abdulaziz University Hospital, Jeddah 21589, Saudi Arabia; maalyousef@kau.edu.sa; 13 Department of Medical Laboratories, College of Applied Medical Sciences, Qassim University, Buraydah 52571, Saudi Arabia; f.alhumaydhi@qu.edu.sa; 14 Centre of Medical and Bio-Allied Health Sciences Research, Ajman University, Ajman P.O. Box 346, United Arab Emirates; 15 Centre for Interdisciplinary Research in Basic Sciences, Jamia Millia Islamia, Jamia Nagar, New Delhi 110025, India

**Keywords:** MARK4, drug repurposing, drug discovery, virtual screening, molecular docking, MD simulation, Alzheimer’s disease, type 2 diabetes mellitus

## Abstract

Microtubule affinity regulating kinase 4 (MARK4) regulates the mechanism of microtubules by its ability to phosphorylate the microtubule-associated proteins (MAP’s). MARK4 is known for its major role in tau phosphorylation via phosphorylating Ser^262^ residue in the KXGS motif, which results in the detachment of tau from microtubule. In lieu of this vital role in tau pathology, a hallmark of Alzheimer’s disease (AD), MARK4 is a druggable target to treat AD and other neurodegenerative disorders (NDs). There is growing evidence that NDs and diabetes are connected with many pieces of literature demonstrating a high risk of developing AD in diabetic patients. Metformin (Mtf) has been a drug in use against type 2 diabetes mellitus (T2DM) for a long time; however, recent studies have established its therapeutic effect in neurodegenerative diseases (NDs), namely AD, Parkinson’s disease (PD) and amnestic mild cognitive impairment. In this study, we have explored the MARK4 inhibitory potential of Mtf, employing in silico and in vitro approaches. Molecular docking demonstrated that Mtf binds to MARK4 with a significant affinity of −6.9 kcal/mol forming interactions with binding pocket’s critical residues. Additionally, molecular dynamics (MD) simulation provided an atomistic insight into the binding of Mtf with MARK4. ATPase assay of MARK4 in the presence of Mtf shows that it inhibits MARK4 with an IC_50_ = 7.05 µM. The results of the fluorescence binding assay demonstrated significant binding of MARK4 with a binding constant of 0.6 × 10^6^ M^−1^. The present study provides an additional axis towards the utilization of Mtf as MARK4 inhibitor targeting diabetes with NDs.

## 1. Introduction

Diabetes is a chronic and common disease that can occur throughout life. It occurs either when sufficient insulin is not released from the pancreas or when the body gets desensitized to insulin and fails to use it effectively. The number of people affected from diabetes is increasing drastically, and the disease also lays foundation for the development of other life threating conditions, such as Alzheimer’s disease (AD), diabetic neuropathy, nephropathy, cardiovascular diseases (CVDs) and various types of cancer, such as liver, pancreas, colon, etc. [1,2,3]. The rate of the development of cancer is much higher in type 2 diabetes mellitus (T2DM) patients when compared to normal individuals. The reason can be resistance to insulin and mitogenic effects of hyperglycaemia [4].

Metformin (*N*,*N*-dimethylbiguanide, Mtf), a guanidine derivative, is extensively used for T2DM and hyperglycaemia from past many decades [5]. The popularity of the drug is due to its low medication cost and high tolerance capability. Another important asset of Mtf is that it can cross the blood–brain barrier and has a potent insulin-sensitizing property, thus it has the potential to treat AD and other related pathologies [6]. Mtf has been studied laboriously for its effect as an anti-diabetic drug by exploiting the molecular machinery of the cell [7]. Mtf has been found to impede various molecular signalling pathways involved in glucose homeostasis and gluconeogenesis. Some of the pathways accessed by Mtf involves the activation of adenosine 5′-monophosphate (AMP)-activated protein kinase (AMPK) through escalating Thr-172 phosphorylation [8]. Mtf also finds its use in reproductive biology to treat cases of polycystic ovary syndrome (PCOS) and obese male fertility as the pathology of the disease is often related with insulin resistance [9,10]. A recent study reported that in the brains of Parkinson’s disease (PD) patients, Mtf causes a reduction in the neuronal damage via neuroprotection and oxidative stress inhibition and inflammatory responses, furnishing a novel PD strategy [11]. Another piece of literature established that long-term Mtf therapy (>2 years) results in the lower incidences of NDs in elderly T2DM patients [12].

Protein kinases are known to modulate almost all of the cellular machinery in a direct or an indirect way. It mediates its function of phosphorylation of targeted protein, and hence controls many important signalling pathways. The roles of various protein kinases on diabetes models have been well demonstrated, which includes AMPK, IκB kinase and protein kinase C (PKC) [13,14,15]. The kinases impede in the tolerance levels of glucose, elevate inflammation and modulate insulin resistance in the cells by acting on targets downstream in the signalling pathways. Microtubule affinity regulatory kinases (MARKs) represent the mammalian homologs of nematode par-1. MARK4 is an important kinase that plays a vital role in disease therapeutics ranging from NDs to diabetes due to various important roles performed by this kinase. MARK4 inhibition enhances glucose homeostasis significantly by up regulation of 5′ AMP-activated protein kinase (AMPK) in tissues [16]. MARK4 plays an important role in energy metabolism and regulation, making the drug as an irrefutable target for treatment of T2DM. Thus, understanding the fact that how kinases are involved in diabetes is of utmost importance. The role of Mtf as an anti-diabetic drug is well-established; it can access several pathways to exhibit this effect. The overexpression of MARK4 contributes to the pathology of AD as it results in hyperphosphorylation of tau and these hyperphosphorylated tau results in the formation of neurofibrillary tangles (NFTs), a major hallmark of AD, thus signifying the importance of MARK4 in AD. There is growing evidence for the association of NDs and diabetes and also for use of Mtf in NDs. However, the exact mechanism of the neuroprotective activity of Mtf is not fully understood. All the above studies show that MARK4 plays a role in diabetes and AD, and thus exploring the MARK4 inhibitory potential of the commonly used antidiabetic drug, Mtf, can open up new domain that is still unexplored. Thus, this study explored the MARK4 inhibitory potential of Mtf using various computational and experimental approaches and will provide a platform to decipher the association of diabetes and neurodegenerative disorders (NDs).

## 2. Results and Discussion

### 2.1. Molecular Docking Analysis

**Figure 1:**
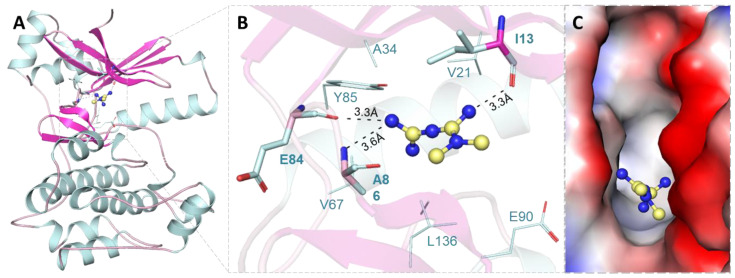
Binding prototype of MARK4 with Mtf. (**A**) Cartoon representation of the protein–ligand complex. (**B**) Magnified view of the Mtf docked pose on MARK4. (**C**) Binding pocket cavity representation of MARK4 occupied with Mtf.

The molecular docking of Mtf was performed by MARK4 to explore their probable binding sites and affinity. Log-file and out-file were generated containing affinity scores and docked poses for Mtf after the docking process. The docking result showed that Mtf has an appreciable binding affinity score, i.e., −6.9 kcal/mol with MARK4. We have observed that Mtf has a preferable interaction with the binding pocket of MARK4 (Figure 1A). The binding mode and interaction pattern of Mtf was analysed utilizing PyMOL, where we visualized the hydrogen bonding and other interactions within the protein–ligand docked complex. It was observed that the residues of the kinase domain of MARK4 offered a significant number of interactions and formed hydrogen bonds with Ile13, Glu84 and Ala86 (Figure 1B). Mtf is docked into the deep binding cavity of MARK4 (Figure 1C). The docking results suggested that Mtf can be an attractive binding partner of MARK4.

**Figure 2:**
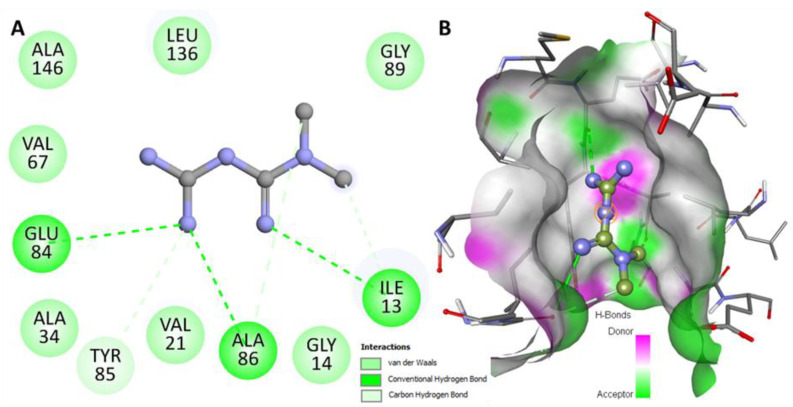
Detailed interactions between MARK4 and Mtf. (**A**) 2D plot of the protein–ligand interactions. (**B**) Mtf binding pocket showing different interactions.

Mtf forms several other hydrophobic interactions with the binding site residues of MARK4 (Figure 2A). It shows a virtuous complementarity with the binding pocket (Figure 2B). These observations clearly suggest a significant binding affinity of Mtf to MARK4.

### 2.2. Structural Changes and Analyses Post MD

**Figure 3:**
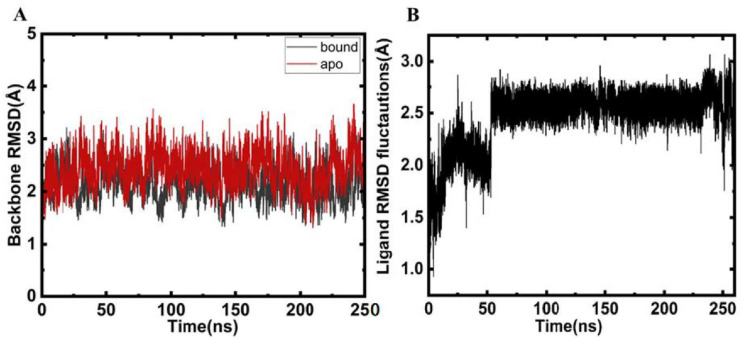
(**A**) RMSD plots of backbone atoms during the 250 ns MD runs. (**B**) RMSD plot of ligand during the 250 ns run.

When small molecules bind to proteins, it leads to significant conformational fluctuations in the native conformation of the protein. To gain an insight into these fluctuations atomistically, MD simulations play a critical role and are at the heart of studies deciphering the conformational alterations in the protein post ligand binding. In a bid to investigate residue-based fluctuations in proteins, root mean squared deviations (RMSD) are a conventional parameter. We calculated the RMSD of apo MARK4 and Mtf-bound MARK4 to decipher the effect of Mtf binding on the conformational dynamics of MARK4. It is apparent from Figure 3 that no significant changes were observed in RMSD of ligand bound and apoprotein. The RMSD plots demonstrate clearly that the protein–ligand complex is stable throughout the 250 ns trajectory implying that binding of ligand does not lead to major change and suggesting the stability of the protein–ligand complex. The RMSD value of ligand bound protein is slightly lower as compared to apoprotein reinforcing the fact that Mtf binding slightly stabilizes the protein as evident from the red RMSD trajectory of the bound protein as compared to the apo form.

**Figure 4:**
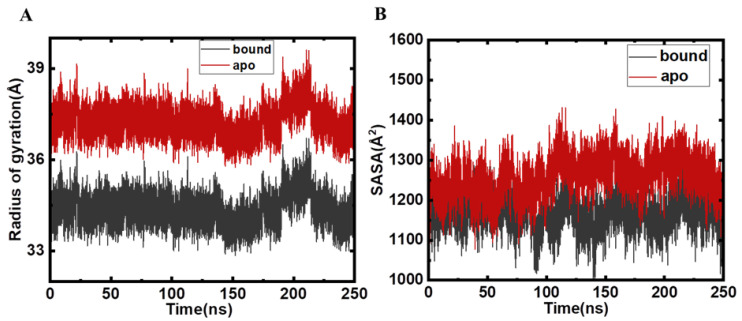
(**A**) *R_g_* plotted during the 250 ns MD run of apo (red) and Mtf-bound MARK4 (black). (**B**) SASA plot of protein backbone atoms as a function of number of frames in the production runs.

The radius of gyration (*R_g_*) is the parameter associated with the tertiary structure of protein and tells about their folding compactness. The determination of *R_g_* was done on the basis of time scale for MARK4 before and after Mtf binding. *R_g_* of the apo and bound MARK4 are depicted in Figure 4A. After an assessment of both systems, the plot was generated, which indicates that MARK4 in the presence of the Mtf shifted towards more compactness during the simulation trajectory.

Solvent accessible surface area (SASA) is directly related to stability of proteins, and it has been used for exploring various conformational microstates in biomolecules. It is a decisive factor that plays a key role in understanding the structural dynamics of the protein after the binding of small molecules. The speedy and precise estimation of SASA aids the energetic analysis of biomolecules [17]. SASA relates to the protein surface area exposed to solvent molecules and is regularly utilized to decipher artefacts in MD simulations of proteins and investigate the changes observed in the conformational dynamics of the proteins after the binding of ligands. During runs, SASA should be comparatively stable with no major deviations observed during the simulation course run. It is quite apparent that the SASA plots of the apo and the bound MARK4 are almost identically implying the stability of protein–ligand complex and delineating the fact that the binding of Mtf with MARK4 stabilises the MARK4 structure (Figure 4B). It is apparent that for the Mtf-bound MARK4 there is a slight increase in SASA values that is attributable to the fact that once Mtf binds to MARK4, the number of internal residues might be exposed to the surface.

### 2.3. Hydrogen Bond Analysis

**Figure 5:**
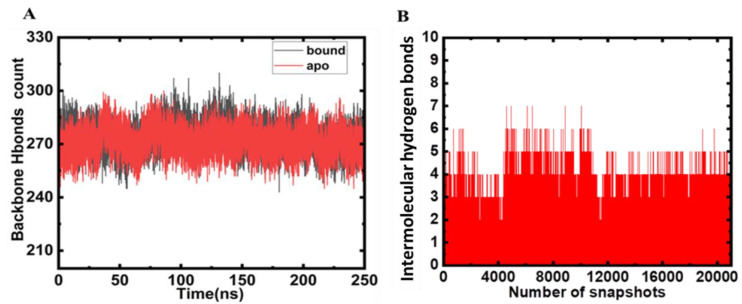
**Hydrogen bonding.** (**A**) Intramolecular hydrogen bonds (both apo and Mtf bound MARK4) plotted as a function of time. (**B**) The time evolution of intermolecular hydrogen bonds monitored between MARK4 and Mtf.

In a bid to maintain the structural conformation of the protein, hydrogen bonds play a pivotal role [18]. The analysis of hydrogen bonding aids in assessment of the stability of the protein and protein–ligand complexes [19]. Thus, herein, we analysed the intramolecular hydrogen bonding to decipher the stability of the MARK4 and MARK4–Mtf complex (Figure 4A). The hydrogen bonds (backbone) formed during Mtf binding and apo protein do not show huge deviations, and they appear to be consistent (Figure 5A). It is apparent that several hydrogen bonds are formed within the protein that is responsible for maintaining the integrity of protein’s three-dimensional structure. Intermolecular hydrogen bonds serve an important criteria for the stability and directionality of the protein–ligand complex [18]. The strength of binding of ligand to the protein can be monitored by measuring the intermolecular hydrogen binds as these give a clue about the strength of the ligand towards the protein’s binding pocket.

During simulation, we calculated the ligand hydrogen bonds and it was estimated that there are 6–7 main hydrogen bonding interactions that appear consistently throughout the 250 ns run, implying the strength of this interaction. Asp-147, Asn-134, Glu-133, and Glu-90 are main hydrogen bond acceptors with donor hydrogen from Mtf. The hydrogen bonds between Mtf and MARK4 are plotted in Figure 5B. This analysis clearly depicted the key role played by hydrogen bonds during this interaction process and highlighting the importance of intermolecular hydrogen bonding in the formation of a stable protein–ligand complex.

### 2.4. Free Energy Analyses

**Figure 6:**
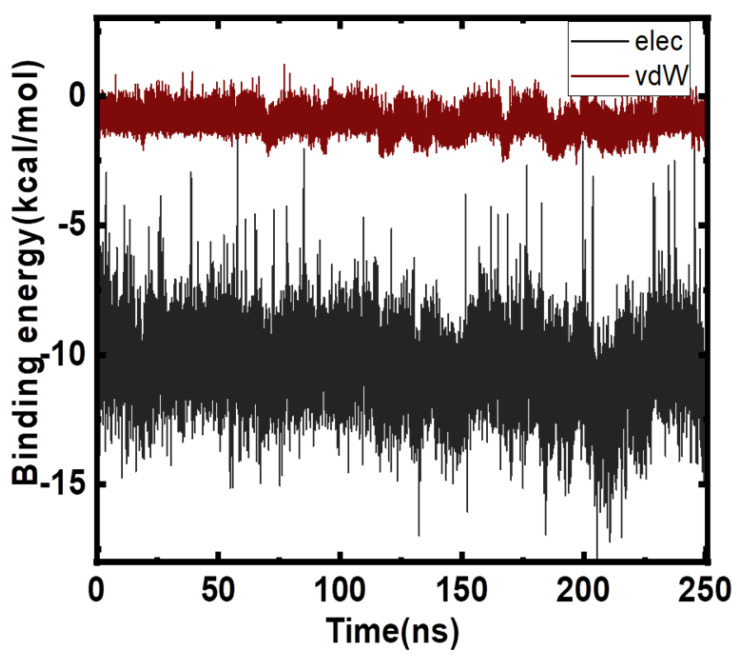
LIE energies plotted as a function of snapshots.

The estimation of the binding affinity of a molecule with target protein is attracting interest of computational chemists. MD simulation studies are becoming increasingly popular to focus on ensemble-based methods that are vital to get statistically significant estimates of free energies. Herein, we employ molecular mechanics/generalized born surface area (MMGBSA) technique and linear interaction energy (LIE) methods as these are very rapid and less demanding approaches. These approaches have been used increasingly to demonstrate structural stability, and to estimate binding affinities and hotspots. MM/GBSA also aids in drug discovery as it allows analysis of the contributions from specific residues or energy terms by free energy decomposition analysis, and also dominant interactions are deciphered in the binding process. The electrostatics and van der Waals interaction energies were computed for the apo protein and for the Mtf-bound form. The net difference in electrostatic and van der Waals components (Mtf-bound Apo Form) is plotted in Figure 6. The net Δ*G*_binding_ was computed as −19.6 kcal/mol. We used Amber Tools to post process the trajectory for calculation of the MMGBSA and PBSA energy estimates. We needed to provide the solvated Mtf-bound–MARK4 complex, dry complex (no waters), protein, and ligand topology files to MMPBSA.py script along with the 250 ns trajectory data. It took approximately 10 h to compute the energies using 6 cores. The data are shown in Table 1. The MMGBSA affinity value estimated –12.1/mol (MMGBSA) is in very close agreement with LIE estimate of −13.5 kcal/mol. The MUE is 1.3 kcal/mol, which is within normal error limits.

### 2.5. Fluorescence Based Binding

**Figure 7:**
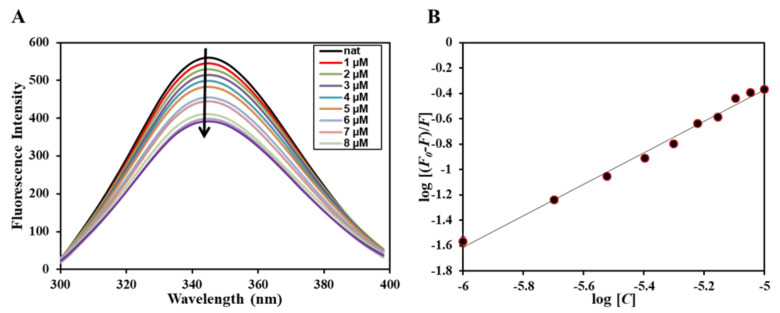
(**A**) Fluorescence emission spectra of MARK4 in the absence and presence of different Mtf concentration (0–10 µM). The protein was excited at 280 nm with emission recorded in the range of 300–400 nm. (**B**) MSV plot obtained for binding of Mtf with MARK4.

Next, after employing in silico approaches and confirming the binding of Mtf to MARK4, we employed fluorescence-based binding to ascertain the actual affinity of the ligand with the protein. It is routinely used to study protein–ligand interactions deciphering various binding parameters that give us a clue regarding the strength of the interaction [20,21]. When a ligand binds to the protein, it results in a decrease in the fluorescence of the native protein and this phenomenon is known as fluorescence quenching. The decrease in the fluorescence of the protein in the presence of the ligand implies that a complex is formed between the protein and the ligand. Figure 7A shows the fluorescence emission spectra of native MARK4 in the absence and presence of varying concentrations of Mtf (0–10 µM), and it is visible that with increasing Mtf concentration, there is a corresponding decrease in the fluorescence intensity of MARK4 signifying that Mtf quenches the fluorescence of MARK4. Further, the obtained quenching data give an idea regarding different binding parameters of the protein–ligand complex revealing the strength of interaction. The quenching data are mathematically evaluated using modified Stern–Volmer (MSV) equation to find the binding parameters; Figure 7B shows the obtained data fitted into MSV equation and the plot obtained is referred to as MSV plot; the intercept of the MSV plot gives the value of binding constant (*K*) that reveals the strength of the interaction of the ligand with the protein. Mtf binds to MARK4 with a binding constant (*K*) of 0.6 × 10^6^ M^−1^. This obtained binding constant reveals the significant strength of the interaction of Mtf with MARK4. Earlier studies have reported other MARK4 inhibitors [22,23], which were also having similar magnitudes of the binding constant suggesting the significance of this interaction and also highlighting the potency of Mtf to act as strong MARK4 inhibitor that will be further evaluated making use of the ATPase assay.

### 2.6. Kinase Assay

**Figure 8:**
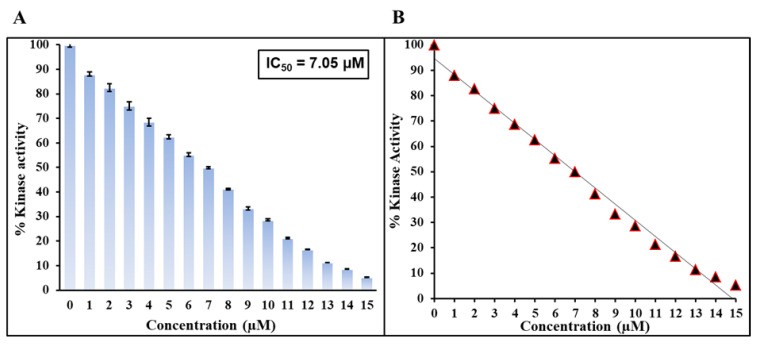
(**A**) ATPase assay of MARK4 with Mtf. The concentration of MARK4 was fixed while Mtf was titrated from 0–15 µM. (**B**) Determination of IC_50_ value of Mtf.

With the aid of computational approaches followed by *in vitro* binding assay, we have confirmed that Mtf shows significant strength of interaction against MARK4 and forms a stable complex. The next aim is to see the effect of Mtf on the functional aspect of MARK4, i.e., how Mtf affects the kinase activity of MARK4. For this, we employed a kinase assay with varying concentrations of the Mtf, as shown in Figure 8. The activity of native MARK4 is taken as 100% for reference, i.e., the activity MARK4 in the absence of Mtf is taken as 100%. It is apparent from the obtained kinase assay profile that with a corresponding increase in the Mtf concentration there is decrease in the kinase activity of MARK4 suggesting the inhibitory effect of Mtf on the kinase activity of MARK4. IC50 is the concentration of ligand/drug required for 50% inhibition [24], i.e., concentration of the inhibitor (ligand) needed to attain 50% inhibition (binding saturation) of the enzyme (receptor). IC_50_ was found to be µM and the value of this order can be correlated with earlier reported MARK4 inhibitors [25,26], thus revealing the fact that Mtf, an antidiabetic drug, inhibits the kinase activity of MARK4. This reinforces the importance of Mtf use in NDs.

## 3. Materials and Methods

### 3.1. Materials

Metformin was purchased from Merck KGaA, Darmstadt, Germany. Ni-NTA resin was purchased from Qiagen for protein purification. N-cyclohexyl-3-aminopropanesulfonic acid (CAPS) buffer was obtained from Himedia (Mumbai, India). NaCl and sodium monophosphate and bisphosphate were obtained from SRL Chemicals (Gurugram, India). All other chemicals used were of analytical grade and double distilled water was used for buffer preparation.

### 3.2. Molecular Docking

We retrieved MARK4 crystal structure from the Protein Data Bank (PDB ID 5ES1) [27]; a high-resolution structure (2.80 Å) without mutations where missing residues were remodelled in PyMod 3. The Mtf structure was downloaded from PubChem with Compound CID: 4091. The docking was performed using InstaDock [28] where search space was defined as −39.46, −14.339, and −1.963 with sizes 50, 70, and 80 for X, Y, and Z coordinates, respectively. All other conditions and parameters were followed as per earlier published literature [29,30].

### 3.3. MD Simulations Setup of the Protein–Ligand Complex

The ligand was washed in MOE2020 prior to docking at pH = 7. MOE platform computed critical descriptors for the ligand (Table 2).

### 3.4. System Preparation Prior to MD Setup

After docking studies, the top pose based on the detailed analysis was selected to initialize MD simulations. To obtain the parameters of the ligand (Mtf), we performed the geometry optimization of the fragment using the B3LYP/6-31G(d) method in the Gaussian 16 program [31]. All other conditions and parameters were followed as per earlier published literature [29,30].

### 3.5. MD Simulation Details

We used GPU-accelerated AMBER20 [32] for MD simulation studies, and then SHAKE [33] was turned with 2 fs timestep. Initially, minimization of 15,000 steps with a 10 kcal/mol. Å^2^ restraint was carried out and the complex was slowly heated from 0 K to 300 K in NVT ensemble in four steps. During this period, protein backbone and ligand atoms were restrained. Post that temperature ramping steps were carried out followed by a fine-tuned elaborate equilibration. A five step equilibration was set up, gradually dialling down the harmonic restraints on the protein backbone from 5, 2, 1, 0.1 kcal/mol Å^2^; at last step there was no limitation. All other conditions and parameters were followed as per earlier published literature [29,30].

### 3.6. Fluorescence Assay

Fluorescence binding studies were carried out on FP-6200 spectrofluorometer (Tokyo, Japan), which is attached to an external water bath that maintains the temperature. We excited the protein at 280 nm and recorded emission in the range of 300–400 nm. The excitation and emission slit widths were set at 10 mm and response was set to medium. All the spectra reported here are the subtracted spectra taking inner filter effect into consideration [34]. Fluorescence data were analysed using modified Stern–Volmer equation (MSV) as per earlier published literature to obtain different binding parameters of MARK4–Mtf complex [35,36].

### 3.7. Kinase Assay

ATPase assay was performed to check the inhibitory effect of Mtf on the kinase activity of MARK4. The activity of MARK4 hinges on the free form of inorganic phosphate (P_i_) that is released during ATP hydrolysis. We checked the effect of Mtf on the ATPase activity of MARK4 using various concentrations of Mtf as in earlier published studies [22]. For this assay, we used malachite green reagent (Biomol, Enzo Life Sciences, New York, NY, USA). We incubated MARK4 (2 µM) with different concentrations of Mtf (0–15 µM) for 1 h at 25 °C. ATP (200 μM) was prepared freshly and we added MgCl_2_ (10 mM) to the reaction mixture and left for incubation for 30 min at 25 °C. Finally, we added BIOMOL^®^ (Kelayres, PA, USA) reagent to terminate the reaction and the green-coloured complex that was formed was read at 620 nm on ELISA MULTISCAN FC reader.

## 4. Conclusions

The present study establishes Mtf as a potent MARK4 inhibitor. In present times, many researchers are attempting to identify modifiable risk factors for AD and other NDs owing to the failure of traditional therapeutics targeting AD and other related pathologies. In the present work, we have employed molecular docking and MD simulation approaches to understand the conformational dynamics and stability of the MARK4–Mtf system. The results clearly suggested that Mtf binds to the active site of MARK4, forming interactions with the critical residues of the binding pocket. Further, the binding of Mtf does not cause any significant alterations in the structure of MARK4, forming a stable MARK4–Mtf complex. Moreover, fluorescence binding assays deciphered the significant affinity of Mtf towards MARK4; Mtf binds to MARK4 with a binding constant of 0.6 × 10^6^ M^−1^. Kinase assay revealed Mtf as a potent MARK4 inhibitor with IC_50_. Mtf is a very promising candidate for drug design and development targeting AD therapeutics and other NDs owing to its various multi-directional properties and safety and pharmacokinetic profile, highlighting the importance of this work.

## Figures and Tables

**Table 1:** MMGBSA free energy estimate for Mtf binding.

Energy Factors	Average	Standard Deviation	Std Error of Mean
VDWAALS	−28.8312	5.9959	0.1971
EEL	34.7349	33.1069	1.0885
EGB	−14.5293	32.5593	1.0705
ESURF	−3.5261	0.6543	0.0215
Δ*G*_gas_	5.9037	31.4944	1.0355
Δ*G*_solv_	−18.0555	32.7427	1.0766
Δ*G* (total)	−12.1517	4.7443	0.1560

**Table 2:** Critical descriptors of the ligand.

Mol Weight	H Bond Donors	H Bond Acceptors	No of Single Rotatable Bonds	No of Aromatic Rings	LogS	Flexibility Index	Lipinski Drug Like Test
130.1	1	0	1	0	1.2	1.4	1

## Data Availability

All the data have been provided in the manuscript.
