# Correction: Ashraf et al. Inhibition of Microtubule Affinity Regulating Kinase 4 by Metformin: Exploring the Neuroprotective Potential of Antidiabetic Drug through Spectroscopic and Computational Approaches. *Molecules* 2022, *27*, 4652

**DOI:** 10.3390/molecules28020600

**Published:** 2023-01-06

**Authors:** Ghulam Md. Ashraf, Debarati DasGupta, Mohammad Zubair Alam, Saleh S. Baeesa, Badrah S. Alghamdi, Firoz Anwar, Thamer M. A. Alqurashi, Sharaf E. Sharaf, Waleed Al Abdulmonem, Mohammed A. Alyousef, Fahad A. Alhumaydhi, Anas Shamsi

**Affiliations:** 1 Department of Medical Laboratory Sciences, College of Health Sciences, and Sharjah Institute for Medical Research, University of Sharjah, Sharjah 27272, United Arab Emirates; 2 College of Pharmacy, University of Michigan, 428 Church Street, Ann Arbor, MI 48109, USA; 3 Pre-Clinical Research Unit, King Fahd Medical Research Center, King Abdulaziz University, Jeddah 21589, Saudi Arabia; 4 Department of Medical Laboratory Sciences, Faculty of Applied Medical Sciences, King Abdulaziz University, Jeddah 21589, Saudi Arabia; 5 Division of Neurosurgery, College of Medicine, King Abdulaziz University, Jeddah 21589, Saudi Arabia; 6 Department of Physiology, The Neuroscience Research Unit, Faculty of Medicine, King Abdulaziz University, Jeddah 21589, Saudi Arabia; 7 Department of Biochemistry, Faculty of Science, King Abdulaziz University, Jeddah 21589, Saudi Arabia; 8 Department of Pharmacology, Faculty of Medicine, King Abdul-Aziz University, Rabigh 21589, Saudi Arabia; 9 Pharmaceutical Chemistry Department, College of Pharmacy, Umm Al-Qura University, Makkah 21955, Saudi Arabia; 10 Clinical Research Administration, Executive Administration of Research and Innovation, King Abdullah Medical City in Holy Capital, Makkah 24246, Saudi Arabia; 11 Department of Pathology, College of Medicine, Qassim University, P.O. Box 6655, Buraydah 51452, Saudi Arabia; 12 Division of Neurosurgery, College of Medicine, King Abdulaziz University Hospital, Jeddah 21589, Saudi Arabia; 13 Department of Medical Laboratories, College of Applied Medical Sciences, Qassim University, Buraydah 52571, Saudi Arabia; 14 Centre of Medical and Bio-Allied Health Sciences Research, Ajman University, Ajman P.O. Box 346, United Arab Emirates; 15 Centre for Interdisciplinary Research in Basic Sciences, Jamia Millia Islamia, Jamia Nagar, New Delhi 110025, India

## Updated Affiliation

The updated affiliation information can be seen in the affiliation part of this correction.

## Missing Funding

In the original publication [1], the funder “Deanship of Scientific Research (DSR), King Abdulaziz University, Jeddah, Saudi Arabia”, **“IFPDP-31-22”** to **“Mohammad Zubair Alam”** was not included. The authors apologize for any inconvenience caused and state that the scientific conclusions are unaffected. The original publication has also been updated.

The corrected Funding and Acknowledgment appear below:
